# Time spent outdoors in childhood is associated with reduced risk of myopia as an adult

**DOI:** 10.1038/s41598-021-85825-y

**Published:** 2021-03-18

**Authors:** Gareth Lingham, Seyhan Yazar, Robyn M. Lucas, Elizabeth Milne, Alex W. Hewitt, Christopher J. Hammond, Stuart MacGregor, Kathryn A. Rose, Fred K. Chen, Mingguang He, Jeremy A. Guggenheim, Michael W. Clarke, Seang-Mei Saw, Cathy Williams, Minas T. Coroneo, Leon Straker, David A. Mackey

**Affiliations:** 1 Centre for Ophthalmology and Visual Science (Incorporating Lions Eye Institute), The University of Western Australia, Perth, Australia; 2 National Centre for Epidemiology and Population Health, Research School of Population Health, Australian National University, Canberra, Australia; 3 Telethon Kids Institute, The University of Western Australia, Perth, Australia; 4 Centre for Eye Research Australia, Department of Ophthalmology and Surgery, University of Melbourne, Royal Victorian Eye and Ear Hospital, Melbourne, Australia; 5 Department of Ophthalmology, Menzies Institute of Medical Research, University of Tasmania, Hobart, Australia; 6 Section of Academic Ophthalmology, School of Life Course Sciences, King’s College London, London, UK; 7 Statistical Genetics Lab, QIMR Berghofer Medical Research Institute, Brisbane, QLD Australia; 8 Discipline of Orthoptics, Graduate School of Health, University of Technology Sydney, Sydney, Australia; 9 State Key Laboratory of Ophthalmology, Zhongshan Ophthalmic Center, Sun Yat-Sen University, Guangzhou, China; 10 School of Optometry and Vision Sciences, Cardiff University, Cardiff, UK; 11 Metabolomics Australia, Centre for Microscopy, Characterisation and Analysis, The University of Western Australia, Perth, Australia; 12 Department of Epidemiology and Public Health, Yong Loo Lin School of Medicine, National University of Singapore, Singapore, Singapore; 13 Department of Population Health Sciences, Bristol Medical School, University of Bristol, Bristol, UK; 14 Department of Ophthalmology, University of New South Wales, Sydney, Australia; 15 School of Physiotherapy and Exercise Science, Curtin University, Perth, Australia; 16 Lions Eye Institute, 2 Verdun St, Nedlands, WA 6009 Australia

**Keywords:** Risk factors, Biomarkers, Epidemiology

## Abstract

Myopia (near-sightedness) is an important public health issue. Spending more time outdoors can prevent myopia but the long-term association between this exposure and myopia has not been well characterised. We investigated the relationship between time spent outdoors in childhood, adolescence and young adulthood and risk of myopia in young adulthood. The Kidskin Young Adult Myopia Study (KYAMS) was a follow-up of the Kidskin Study, a sun exposure-intervention study of 1776 children aged 6–12 years. Myopia status was assessed in 303 (17.6%) KYAMS participants (aged 25–30 years) and several subjective and objective measures of time spent outdoors were collected in childhood (8–12 years) and adulthood. Index measures of total, childhood and recent time spent outdoors were developed using confirmatory factor analysis. Logistic regression was used to assess the association between a 0.1-unit change in the time outdoor indices and risk of myopia after adjusting for sex, education, outdoor occupation, parental myopia, parental education, ancestry and Kidskin Study intervention group. Spending more time outdoors during childhood was associated with reduced risk of myopia in young adulthood (multivariable odds ratio [OR] 0.82, 95% confidence interval [CI] 0.69, 0.98). Spending more time outdoors in later adolescence and young adulthood was associated with reduced risk of late-onset myopia (≥ 15 years of age, multivariable OR 0.79, 95% CI 0.64, 0.98). Spending more time outdoors in both childhood and adolescence was associated with less myopia in young adulthood.

## Introduction

The prevalence of myopia (near- or short-sightedness) is increasing worldwide; in parts of East and South-East Asia 70–90% of young adults have myopia^1–3^. The rising prevalence of myopia will increase the burden of visual impairment from myopia-associated eye conditions such as retinal detachment and myopic maculopathy^4^. Spending more time outdoors reduces the risk of myopia^5–8^ and, to combat the rising prevalence of myopia, some nations have implemented public health interventions that encourage children to spend more time outdoors. The majority of studies^5–8^ on outdoor exposure and myopia have not followed-up participants beyond 5–6 years, thus it is not known whether spending more time outdoors reduces the risk of myopia beyond this timeframe. Furthermore, no longitudinal studies have examined whether outdoor exposure in teenagers (beyond 12–13 years) reduces risk of later-onset myopia, a relevant time period as myopia continues to develop during and after adolescence^9^.

A challenge when investigating the relationship between time outdoors and myopia is the accurate assessment of outdoor exposure. Studies have predominantly relied on self-reported or parent-reported measures, both of which are valid but are often inaccurate and subject to recall bias^10–13^. Some studies have used objective measures of sunlight exposure to assess time spent outdoors, such as light meters^5,14–16^, 25-hydroxyvitamin D [25(OH)D] concentration^17–23^—a measure of vitamin D adequacy—and conjunctival ultraviolet autofluorescence (CUVAF) area^24,25^, but these can be affected by device compliance or positioning, skin type, and use of sun protection^12^.

We aimed to investigate the relationship between past time spent outdoors during childhood, adolescence and young adulthood and myopia status in young adulthood using multiple measures of outdoor exposure.

## Methods

The Kidskin Young Adult Myopia Study [KYAMS; details previously published^26^] is a follow-up of the Kidskin Study, a non-randomised controlled trial that aimed to reduce sun exposure in children through a sun exposure-intervention. In 1995, the Kidskin Study enrolled 1776 children attending their first year of school (aged 5–6 years) in the Perth metropolitan region, Australia. Schools were assigned to a control arm or to a high-intensity (high group) or moderate-intensity (moderate group) intervention arm^27^. The intervention ran between 1995 and 1999 and consisted of varying intensity of school-work, take-home educational material and access to sun-protective swim-wear^27,28^. In 1995, 1997, 1999 and 2001, parents of Kidskin Study participants completed questionnaires reporting their child’s time spent outdoors at the beach, pool or around the home in the previous summer holidays. The number of melanocytic nevi on the backs of child participants was assessed in 1995, 1999 and 2001 according to a standard protocol^27,29^. The Kidskin Study found no significant difference in the development of melanocytic nevi on the back of children between the intervention and control groups^29^; however, parent-reported sun exposure was significantly lower in the intervention groups, compared to the control groups, at the 1997 and 1999 follow-ups (during the intervention) but not at the 2001 follow-up (2 years post-intervention)^30^. The Kidskin Study was approved by the University of Western Australia and Curtin University Human Research Ethics Committees and a parent or legal guardian provided written informed consent prior to their child participating.

The KYAMS ran from May 2015 to March 2019 and aimed to assess the effect of the Kidskin Study intervention and past time spent outdoors on myopia within the Kidskin Study cohort. The study was approved by the University of Western Australia Human Research Ethics Committee (RA/4/1/6807), registered with the Australian New Zealand Clinical Trials Registry (ACTRN12617000812392, registered 02/06/2017) and performed in accordance with the relevant guidelines and regulations. Written informed consent was provided prior to participating. All Kidskin Study participants were eligible to participate and were contacted using multiple methods (letter, phone call, social media)^31^. The examination included autorefraction (Nidek ARK-510A, Nidek Co. Ltd, Japan) ≥ 20 min after instillation of 1 drop of tropicamide 1% and lensometry (CL-200 Computerized Lensmeter, Topcon Medical Systems, Inc, USA). Myopia was defined as:

1. A mean cycloplegic spherical equivalent of both eyes < − 0.50 dioptres (D); OR
2. A self-reported history of refractive surgery and of myopia prior to the surgery; OR
3. If cycloplegic autorefraction was absent, a mean spherical equivalent of both eyes of < − 0.50 D on non-cycloplegic autorefraction and prescription spectacles with a mean refraction < − 0.50 D.

### Measures of time spent outdoors

An overview of the measures of time spent outdoors used in this study is provided in Table 1.

**Table 1:** Overview of measures of time spent outdoors in the Kidskin Study and the KYAMS Study.

Name	Type	Description	Follow-ups
Parent-reported time outdoors (h/day)	Subjective	Participant’s time spent outdoors in the sun over the previous summer holidays as reported by parents of participants^30,36^	1997, 1999, 2001
Melanocytic nevi count of the back (change/year)^48^	Objective	Number of melanocytic nevi on the back. An average yearly change in melanocytic nevi was calculated using baseline data and the latest follow-up for which data were available	1995, 1999, 2001
Self-reported sun calendar (h/day)	Subjective	Time spent outdoors in the sun in leisure time during summer and winter for each year of life from age 5–26 years as reported by participants	KYAMS
Self-reported current (h/day)	Subjective	Time spent outdoors on an average working and non-working day in summer and in winter at time of questionnaire as reported by participants	KYAMS
Serum 25(OH)D concentration (nmol/L)^11,49^	Objective	Serum 25(OH)D concentration as measured by liquid-chromatography tandem mass-spectrometry^33^	KYAMS
CUVAF area (mm^2^)^50–52^	Objective	Sum of CUVAF area measured on the nasal and temporal conjunctiva of the right and left eye using a semi-automated program^32^	KYAMS
Actinic skin damage score^11,53^	Objective	Skin damage score assessed from silicone skin cast taken of the dorsum of the right hand^35^	KYAMS
Number of melanocytic nevi on the right arm^53^	Objective	Number of melanocytic nevi on the right arm including hand	KYAMS

*25(OH)D* 25-hydroxyvitamin D, *CUVAF* conjunctival ultraviolet autofluorescence.

#### Objective measures of time spent outdoors

CUVAF photographs were taken of the nasal and temporal conjunctiva of each eye and the area measured using a validated semi-automated software (MATLAB 2013b, MathWorks, Natick, USA)^32^. Blood samples for 25-hydroxyvitamin D [25(OH)D] were collected from participants on the day of examination and sera analysed for 25(OH)D (sum of 25(OH)D_2_ and 25(OH)D_3_) concentration at the completion of data collection using liquid-chromatography tandem mass-spectrometry (LC/MS–MS), calibrated according to a standard reference^33^. Actinic skin damage score was assessed by a single experienced examiner^34^ from silicone skin casts taken from the right hand of participants using a 6-grade scale^35^. The number of melanocytic nevi on the right arm of participants was counted by examiners according to the KYAMS protocol. Change in number of melanocytic nevi/year on the backs of Kidskin Study participants between 1995 and 2001 (or 1999 if 2001 data not available) was also used as an objective measure of time spent outdoors in this study^29^.

#### Subjective measures of time spent outdoors

Parent-reported average daily time spent outdoors in the previous summer holidays was derived from Kidskin Study questionnaires completed by parents at the 1997, 1999 and 2001 follow-ups (when participants were aged approximately 8, 10 and 12 years) using the previously reported method^36^. The wording of the Kidskin Study questionnaires differed slightly across follow-ups; for this reason, an average daily parent-reported time spent outdoors could not be calculated for the 1995 baseline and was not included in this analysis^30,36^.

In the KYAMS, participants additionally self-reported two measures of time spent outdoors: a current time spent outdoors and a sun calendar time spent outdoors. Current time spent outdoors was reported as a continuous variable for leisure time on working and non-working days in summer and in winter. An average daily current time spent outdoors was calculated as (2 × non-working day) + (5 × working day)/7 for summer and winter and the mean of this calculated to obtain a total daily current time spent outdoors. In the sun calendar, participants reported their average daily time spent outdoors in the sun in summer and in winter leisure time for each year of life from age 5 to 26 years. Sun calendar responses were categorical and response options were (0–0.5 h, 0.5–1 h, 1–2 h, 2–3 h, 3–4 h and > 4 h). Ages were grouped into 5–9, 10–14, 15–19 and 20–26 years. An average time spent outdoors was calculated for each age group by assigning each categorical time spent outdoors response a numeric value (0.25, 0.75, 1.5, 2.5, 3.5 and 5 h, respectively) and calculating the average time spent outdoors (mean of summer and winter) for each year, then calculating the mean of all years in each age group.

### Covariate data

Covariate data were largely derived from questionnaire measures. Parental education was reported by parents during the Kidskin Study. Parental education was labelled as ‘tertiary’ if either caregiver had obtained a tertiary qualification. KYAMS participants self-reported their highest level of education achieved (primary school, secondary school, apprenticeship, vocational, undergraduate, postgraduate) and this was collapsed into university vs non-university. KYAMS participants self-reported their parent’s ancestry and ancestry was coded as “European” if both parents were reported as being of European ancestry. Occupation was self-reported according to divisions of the Australian and New Zealand Standard Industrial Classification (2006). Based on the findings of a previous Western Australia study^37^, participants who reported working in the agriculture, forestry and fishing, mining, electricity, gas, water and waste, construction, transport, postal and warehousing, rental, hiring and real estate service or “other services” were classified as having outdoor occupations and everyone else was classified as having an indoor occupation, including those caring for their child. Participants reported whether their mother, father or caregiver was short-sighted.

### Statistical analysis

#### Relationships between measures of sun exposure

We investigated the relationships between each of the measures of time spent outdoors using univariable regression modelling: ordinal logistic regression when skin damage score was the outcome; negative binomial regression when nevus count (back or arm) was the outcome; and linear regression for continuous non-count outcomes. Serum 25(OH)D concentration was de-seasonalized as described in the Supplementary Information prior to analysis.

We developed index measures of time spent outdoors in the sun using factor analysis. Factor analysis identifies one or more factors (underlying latent variables) that explain the maximum possible amount of the variance and covariance in the observed measures of time spent outdoors (indicators). We used confirmatory factor analysis (CFA) to generate factor scores that represent the relative ranking of participants within the factor. CFA requires user specification of the model structure but allows formal testing for model improvement or for correlations between indicators and factors and can handle missing data when using full information maximum likelihood estimation. The methods for specifying the CFA model are described in the supplementary material. Factor scores were generated from the final CFA models using the regression method, which produces normally distributed factor scores that range between − 1 and 1 and have a mean of 0.

#### Association between intervention group, time spent outdoors and myopia

The primary and secondary outcomes were myopia status and cycloplegic spherical equivalent at the KYAMS, respectively. We investigated the association between each outcome and: (1) Kidskin Study intervention group; (2) individual measures of time spent outdoors; and (3) index variables of time spent outdoors. Logistic and linear regression were used to assess associations when myopia or spherical equivalent was the outcome, respectively. Data from three individuals with moderate- to high-hyperopia (spherical equivalents: + 4.9, + 6.7 and + 7.9 D) were excluded as outliers when spherical equivalent was the outcome.

Potential confounders were included in multivariable analyses. Age, sex, education (university degree vs no university degree), number of parents with myopia (none, one or two), ancestry (European vs non-European) and intervention group were identified as potential confounders prior to analysis. Other potential confounders were included if p < 0.10 on univariable testing with either outcome. Analyses were conducted in R v3.5.1 (R foundation for statistical computing, Vienna, Austria, https://www.R-project.org). The significance level was 5% and confidence intervals [CI] are 95%.

## Results

### Demographics

Characteristics of the 303 KYAMS participants are shown in Table 2. The prevalence of myopia was 29.4%. Compared to the baseline Kidskin Study participants, KYAMS participants were more likely to be female, in an intervention group, and have a parent with a tertiary education^31^. Additional confounders identified were indoor vs outdoor occupation and parental education (indoor vs outdoor occupation: mean difference = − 0.78 D, p < 0.001; tertiary vs non-tertiary: mean difference = − 0.42 D, p = 0.06). Supplementary Table 1 provides descriptive statistics for measures of time spent outdoors.

**Table 2:** Demographics of participants of the Kidskin Young Adult Myopia Study.

	No myopia (n = 214)	Myopia (n = 89)	Total (n = 303)	*P* value^a^
Mean age (range)	27.5 (25.6, 30.0)	27.6 (25.3, 29.9)	27.5 (25.3, 30.0)	0.86
**Sex, n (%)**				0.09
Male	89 (76.7%)	27 (23.3%)	116 (38.3%)	
Female	125 (66.8%)	62 (33.2%)	187 (61.7%)	
**Caucasian**				0.42
Yes	182 (71.9%)	71 (28.1%)	253 (87.5%)	
No	23 (63.9%)	13 (36.1%)	36 (12.5%)	
**Intervention group**				0.96
Control	71 (69.6%)	31 (30.4%)	102 (33.7%)	
Moderate	79 (71.2%)	32 (28.8%)	111 (36.6%)	
High	64 (71.1%)	26 (28.9%)	90 (29.7%)	
**University degree**				0.82
No	97 (71.9%)	38 (28.1%)	135 (46.9%)	
Yes	107 (69.9%)	46 (30.1%)	153 (53.1%)	
**Occupation**				0.10
Indoor	133 (67.9%)	63 (32.1%)	196 (68.3%)	
Outdoor	71 (78.0%)	20 (22.0%)	91 (31.7%)	
**Number of parents with myopia**				0.02
Zero	129 (77.2%)	38 (22.8%)	167 (57.8%)	
One	53 (63.9%)	30 (36.1%)	83 (28.7%)	
Two	23 (59.0%)	16 (41.0%)	39 (13.5%)	
**Highest parental education**				0.20
Non-tertiary	119 (74.4%)	41 (25.6%)	160 (53.5%)	
Tertiary	93 (66.9%)	46 (33.1%)	139 (46.5%)	

14 individuals were missing all questionnaire data.

^a^t test or chi square test where applicable.

### Relationship between sun exposure variables

Supplementary Table 2 shows the relationships between individual measures of outdoors exposure on univariable analysis. Recall of time spent outdoors between ages 5–14 years in the KYAMS and nevus count of the right arm were found to be of limited value as measures of past time outdoors and excluded from further analyses^36^.

Recall of time spent outdoors between ages 15–19 years and 20–26 years were highly correlated (Pearson r = 0.83, 95% CI 0.79, 0.86) and were therefore collapsed together. Only 126 (41.6%) participants had complete data on the remaining nine measures of time spent outdoors. However, 214 (70.3%) participants were missing only one or fewer measures and 271 (89.4%) were missing two or fewer measures of outdoor exposure.

### Development of a sun exposure index

**Figure 1:**
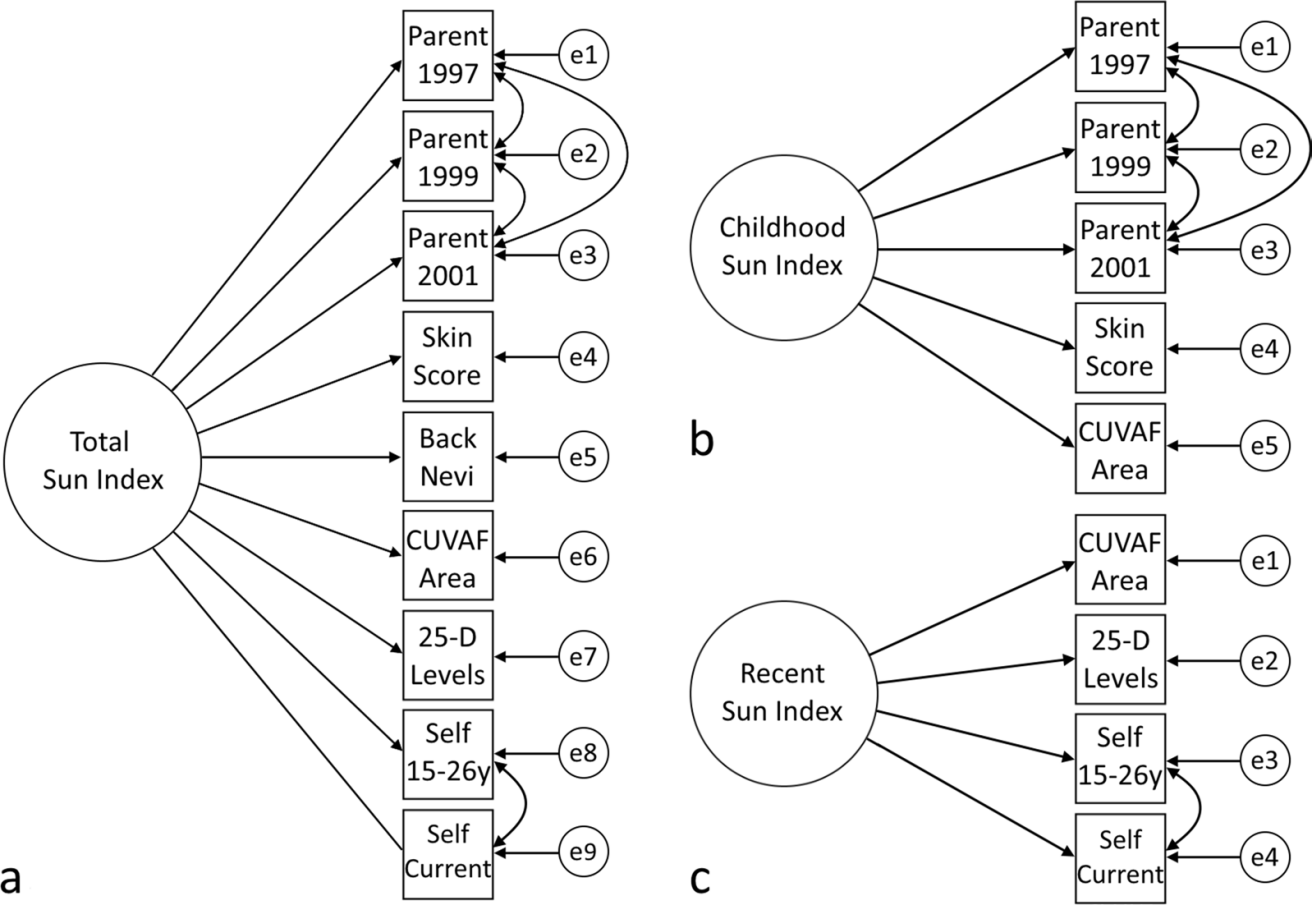
Pathways of the CFA models used to generate factor scores for the total sun exposure index (**a**), childhood sun exposure index (**b**) and recent sun exposure index (**c**). Double-headed arrows indicate covariance terms; small circles indicate error terms. Parent: Parent-reported time spent outdoors. Self: self-reported time spent outdoors. *25-D* 25-hydroxyvitamin D, *CUVAF* conjunctival ultraviolet autofluorescence, *Y *years, *Back nevi* average yearly change in number of nevi on the backs between ages 6–12 years, *Skin score* skin damage score.

**Figure 2:**
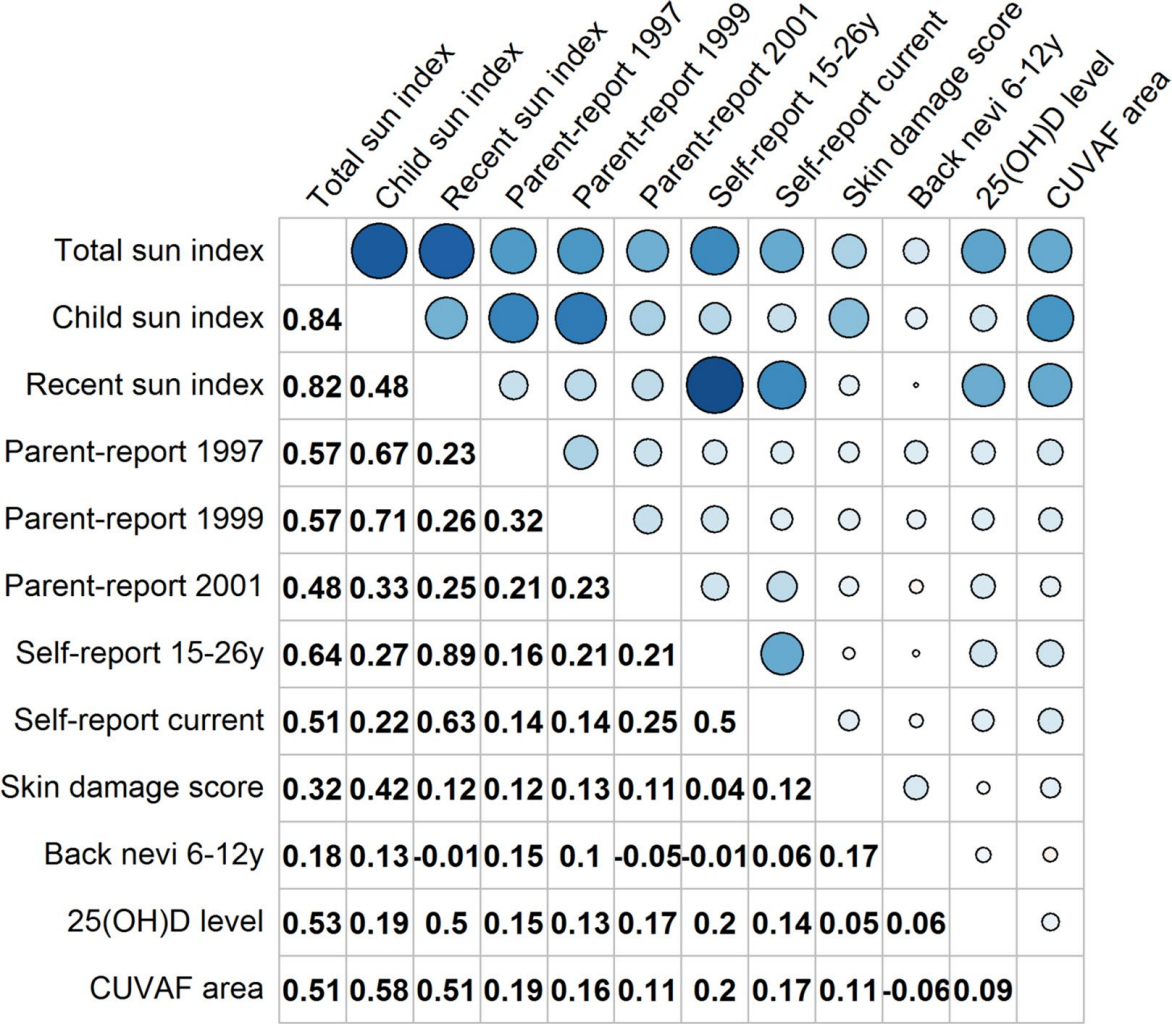
Correlation between each of the sun-exposure indices and observed measures of sun exposure. Pearson correlation coefficient is presented in lower half of table; correlations involving skin damage score are the polyserial correlation coefficient due to the ordinal nature of skin damage score. In the upper half, the size and darkness of the circles represent the strength of the correlation with larger and darker circles representing a higher correlation coefficient. All correlations ≥ 0.13 are significantly greater than 0 (i.e. p < 0.05). *25(OH)D* 25-hydroxyvitamin D, *CUVAF* conjunctival ultraviolet autofluorescence. Figure drawn using the ‘corrplot’ package in R v3.5.1 (R foundation for statistical computing, Vienna, Austria, https://www.R-project.org).

Exploratory factor analysis indicated that the measures of time spent outdoors were best described by a two-factor model, one factor representing childhood time outdoors and the other more recent time outdoors. However, the two factor scores were highly correlated (Pearson r = 0.90) and thus had limited value as separate measures of outdoor exposure. For practical reasons, we therefore specified a one-factor model (Fig. 1a) representing total past time spent outdoors (total sun index)^38^. To investigate the impact of spending time outdoors in childhood or later adolescence/young adulthood on myopia outcomes, we separately specified two one-factor models designed to assess childhood time spent outdoors in the sun (childhood sun index, Fig. 1b) and recent time spent outdoors in the sun (recent sun index, Fig. 1c), respectively. All models had adequate goodness of fit statistics (p > 0.05, comparative fit index > 0.95, root-mean-square error of approximation < 0.05, standardised root-mean-square residual < 0.05) and the standardized factor loadings and R-square statistics are shown in Supplementary Table 3. The correlations between the sun indices (factor scores) and the indicator measures of time spent outdoors are shown in Fig. 2.

### Relationship between intervention group and myopia

The prevalence of myopia was 30.4%, 28.8% and 28.9% in the control, moderate and high intervention groups, respectively. Compared to the control group, there was no significant association between intervention group and prevalence of myopia or spherical equivalent (Supplementary Table 4).

### Relationship between time outdoors and myopia: individual measures of time outdoors

Table 3 and Supplementary Table 4 show the associations between the outcomes and individual measures of time spent outdoors on univariable and multivariable analysis, respectively. Greater parent-reported time spent outdoors during childhood and larger CUVAF area were associated with reduced myopia risk. Greater self-reported time outdoors at the time of the KYAMS and larger CUVAF area were similarly associated with a more hyperopic spherical equivalent.

**Table 3:** Associations between myopia or spherical equivalent and individual measures of time spent outdoors using multivariable regression.

	Myopia^a,b^			Mean Spherical Equivalent^a,c,d^		
	Odds ratio (95%CI)	p	n	Beta (95% CI)	p	n
**Intervention group**			282			263
Control	Reference			Reference		
Moderate	0.81 (0.42, 1.56)	0.53		0.18 (− 0.33, 0.68)	0.50	
High	0.84 (0.42, 1.65)	0.61		0.16 (− 0.36, 0.68)	0.54	
**Time spent outdoors (h/day)**						
Parent-reported (8 years)	0.98 (0.81, 1.19)	0.88	253	− 0.01 (− 0.16, 0.14)	0.88	235
Parent-reported (10 years)	0.71 (0.56, 0.91)	< 0.01	252	0.18 (0.03, 0.33)	0.02	236
Parent-reported (12 years)	0.82 (0.67, 1.01)	0.06	240	0.11 (− 0.02, 0.25)	0.10	226
Parent-reported (mean of 8–12 years)	0.75 (0.57, 0.98)	0.03	276	0.16 (− 0.03, 0.35)	0.10	258
Self-reported KYAMS sun calendar (mean 15–26 years)	0.88 (0.65, 1.18)	0.38	239	0.18 (− 0.04, 0.41)	0.11	223
Self-reported current time outdoors (KYAMS)	0.82 (0.66, 1.03)	0.09	252	0.18 (0.03, 0.32)	0.01	236
De-seasonalized 25(OH)D per 10 nmol/L increase	1.02 (0.91, 1.15)	0.72	240	0.02 (− 0.07, 0.11)	0.65	226
Total CUVAF area (mm^2^) per 10 mm^2^ increase	0.87 (0.77, 0.98)	0.02	274	0.13 (0.05, 0.22)	< 0.01	256
Skin score per 1 category increase	1.17 (0.87, 1.57)	0.30	275	− 0.14 (− 0.37, 0.09)	0.22	256
Average rate of change of back naevi 6–12 years	1.20 (0.94, 1.52)	0.27	247	0.00 (− 0.18, 0.19)	0.99	229

*25(OH)D* 25-hydroxyvitamin D, *CUVAF* conjunctival ultraviolet autofluorescence.

^a^Adjusted for age, sex, university education, outdoor occupation, parental myopia, parental education, Caucasian/non-Caucasian race and Kidskin Study intervention group.

^b^Logistic regression.

^c^Linear regression.

^d^18 participants missing post-cycloplegic spherical equivalent data (n = 11) or had prior refractive surgery (n = 7) and data from 3 participants with moderate- to high-hyperopia excluded as outliers.

### Relationship between time outdoors and myopia: sun indices

**Figure 3:**
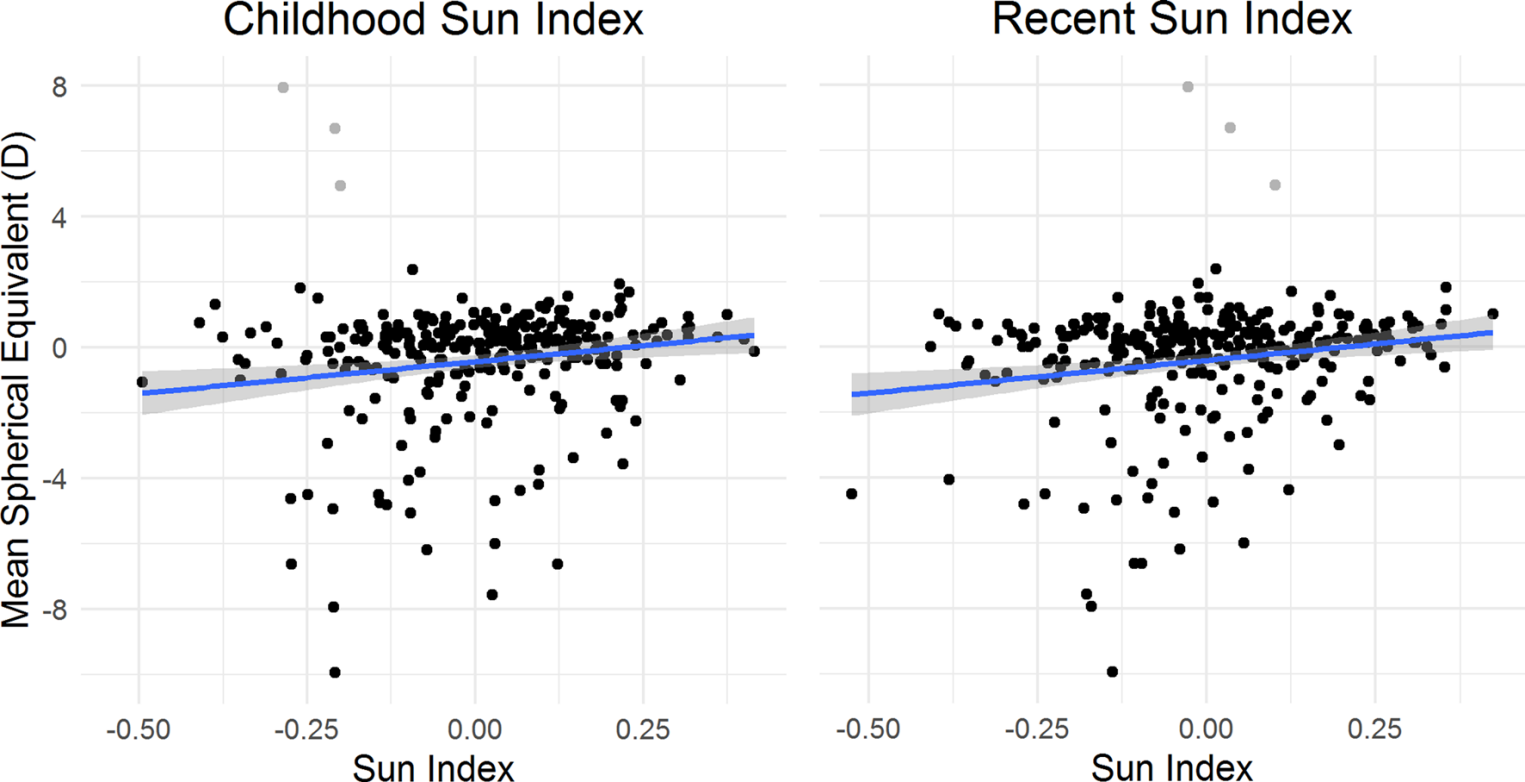
Plots of spherical equivalent over childhood (left) and recent (right) sun indices. The blue line represents the simple linear regression line and grey shading represents 95% confidence intervals. Data from three individuals with moderate to high hyperopia (grey points) were excluded when calculating the linear regression equation and 95% confidence interval. Figure drawn using the ‘ggplot2’ package in R v3.5.1 (R foundation for statistical computing, Vienna, Austria, https://www.R-project.org).

The associations between the sun indices and myopia are shown in Table 4 and plotted in Fig. 3. A higher (i.e. more time spent outdoors) childhood sun index was associated with a lower risk of myopia at the KYAMS and a higher total or recent sun index were associated with a more hyperopic refractive error, after adjustment for confounders. Adjustment for parental myopia, parental education and sex had the greatest impacts on the estimate of the association between the sun indices and myopia. We used self-reported age when first prescribed refractive correction to investigate whether the effect of childhood or recent time spent outdoors varied between those with onset of myopia < 15 years or ≥ 15 years of age. None of the sun indices were significantly associated with self-reported myopia onset < 15 years of age (n = 28); however, a higher recent sun index was associated with reduced risk of myopia onset ≥ 15 years of age (n = 47).

**Table 4:** Association between myopia or spherical equivalent and indices of time spent outdoors in the sun.

	Myopia^a^				Spherical Equivalent^b^			
	Univariable (n = 303)		Multivariable^c^ (n = 266)		Univariable (n = 282)		Multivariable^c^ (n = 263)	
	OR (95% CI)	p	OR (95% CI)	p	Beta (95% CI)	p	Beta (95% CI)	p
**All participants (n = 89 with myopia)**								
Total sun index	0.81 (0.70, 0.94)	< 0.01	0.86 (0.73, 1.01)	0.07	0.27 (0.15, 0.39)	< 0.01	0.15 (0.02, 0.27)	0.02
Child sun index	0.79 (0.67, 0.92)	< 0.01	0.82 (0.69, 0.98)	0.03	0.27 (0.14, 0.40)	< 0.01	0.13 (0.00, 0.27)	0.05
Recent sun index	0.84 (0.72, 0.98)	0.02	0.88 (0.74, 1.03)	0.12	0.23 (0.11, 0.36)	< 0.01	0.16 (0.04, 0.28)	0.01
**Myopia onset < 15 years (n = 28) vs no myopia**								
Total sun index	0.75 (0.60, 0.95)	0.02	0.91 (0.68, 1.23)	0.55	0.28 (0.14, 0.41)	< 0.01	012 (− 0.01, 0.26)	0.08
Child sun index	0.73 (0.57, 0.92)	< 0.01	0.84 (0.62, 1.13)	0.25	0.29 (0.15, 0.43)	< 0.01	0.14 (− 0.01, 0.28)	0.07
Recent sun index	0.79 (0.62, 1.01)	0.06	0.89 (0.65, 1.20)	0.44	0.21 (0.07, 0.35)	0.003	0.11 (− 0.02, 0.25)	0.11
**Myopia onset ≥ 15 years (n = 47) vs no myopia**								
Total sun index	0.81 (0.67, 0.98)	0.03	0.80 (0.65, 0.98)	0.03	0.07 (0.02, 0.16)	0.02	0.07 (− 0.003, 0.15)	0.06
Child sun index	0.83 (0.68, 1.02)	0.07	0.82 (0.66, 1.02)	0.07	0.07 (− 0.01, 0.15)	0.10	0.06 (− 0.03, 0.14)	0.18
Recent sun index	0.81 (0.67, 0.98)	0.03	0.79 (0.64, 0.98)	0.03	0.10 (0.03, 0.17)	< 0.01	0.09 (0.01, 0.17)	0.02

One person missing recent sun exposure; three people with moderate- to high-hyperopia were excluded from analyses of spherical equivalent.

All effect sizes per a 0.1 unit increase in factor score.

^a^Logistic regression.

^b^Linear regression.

^c^Adjusted for age, sex, university education, outdoor occupation, parental myopia, parental education, Caucasian/non-Caucasian race and Kidskin Study intervention group.

The sun indices are ranking variables; to assist interpretation, we divided them into quartiles. Compared to the highest quartile, being in the lowest quartile of either the childhood or recent sun index was associated with an approximate doubling in the prevalence of myopia (childhood: 40.8% vs 19.7%; recent: 38.2% vs 19.7%) and was also associated with approximately 2 h less reported time spent outdoors in the summer holidays at 8, 10 and 12 years of age (parent-reported, mean 3.45 vs 1.24 h) or in leisure time between 15 and 26 years (self-reported, mean 2.83 vs 0.93 h), respectively.

### Sensitivity analysis

We re-assessed the association between myopia and the sun indices using only participants with complete data on outdoor exposure (n = 126). Some associations were no longer significant due to lower statistical power but the estimates of the effects of the sun-exposure indices on myopia were approximately similar (Supplementary Table 5).

## Discussion

We found no evidence to suggest that the Kidskin Study sun exposure-intervention impacted long-term risk of myopia in KYAMS participants. Nevertheless, using indices developed from subjective and objective measures of past time spent outdoors in the sun, spending less time outdoors during childhood was associated with a higher risk of myopia in young adulthood and spending less time outdoors during late adolescence and early adulthood was associated with a more myopic refractive error in young adulthood and a lower risk of late onset myopia (≥ 15 years). Finally we found that compared to the highest quartile, being in the lowest quartile of the childhood or recent sun index was associated with an approximate doubling in the prevalence of myopia and with reportedly spending, on average, 2 h less time outdoors daily over the summer holidays in childhood and in leisure time between 15 and 26 years of age.

In previous studies, greater self-reported time spent outdoors, parent-reported time spent outdoors, serum 25(OH)D concentrations, and CUVAF area have all been associated with lower risk of myopia in children, adolescents and/or young adults^7,8,17,24,25^. In our study, greater mean parent-reported time spent outdoors over the summer holidays at ages 8, 10 and 12 years and a larger CUVAF area were associated with lower risk of myopia in young adulthood. More time spent outdoors at age 10 years, more current time spent outdoors (i.e. at the time of the KYAMS examination), and a larger CUVAF area were associated with a more positive spherical equivalent. We did not detect an association between 25(OH)D concentration and myopia in the KYAMS, possibly due to a lack of power or the age of KYAMS participants. Lower 25(OH)D concentration has been associated with higher risk of myopia in participants younger than 22 years^17–19,22^, but this association is not apparent in adults > 45 years^20,21^.

There are several novel aspects to our study. We created indices of time spent outdoors in the sun based on multiple objective and subjective measures. To ensure that an index variable that actually represents time spent outdoors is created using the CFA models, it is essential that the individual indicator measures of time spent outdoors be valid measures. Should these indicator variables actually reflect some other factor then the created index variable will also be a measure of this unrelated factor. We were therefore careful to select only internally and externally validated measures of time spent outdoors for inclusion in the CFA models and only maintained variables in the CFA model if they showed a correlation with the latent index variable. It is therefore very likely that these index variables do assess time spent outdoors and are a better measure of time spent outdoors than any individual indicator measure. Indeed, this may be the reason why we were able to detect significant associations with a relatively small sample size. Future studies may be able to utilize wearable light meters to obtain more accurate measurements of time spent outdoors. Light meters provide snapshots of time spent outdoors often over a short-term period (e.g. 2 weeks) and cannot be used to assess cumulative past time spent outdoors. Thus, while the addition of light meters in this study would likely have improved the index measures of time spent outdoors, it would not have obviated the need to include other measures of time spent outdoors.

To our knowledge, this is the first study to show that time spent outdoors in early life may reduce the risk of myopia in adulthood. We also showed that spending more time outdoors in adolescence and young adulthood was associated with lower risk of late onset myopia, which agrees with previous cross-sectional findings^17,24^, and indicates that the application of public health interventions that increase time spent outdoors in adolescence are likely to lower risk of subsequently developing myopia^7,17,24^. Our results suggest that time spent outdoors through adolescence and early adulthood may modify final refractive error. This could indicate that spending more time outdoors during this adolescence/young adult period can slow the progression of myopia, a finding that has previously been shown^5,39,40^ but remains somewhat contentious^6,41,42^. The average reported time spent outdoors per day was 2 h higher (approximately 3 vs 1 h outdoors/day) in the highest quartile of the recent and childhood sun-exposure indices, compared to the lowest quartile. Public health interventions in both Taiwan and Singapore recommend children spend at least 2 h a day outdoors. Our findings suggest that, if children were to increase time spent outdoors from 1 to 3 h each day, their risk of myopia could be reduced by 50%; however, this does not account for the many other complex behavioural factors and interactions that could impact myopia risk.

This study has some limitations. First, KYAMS participants were not representative of Kidskin Study baseline participants. Thus, attrition bias could influence our investigation of the effect of intervention group on myopia prevalence. Second, we relied on self-reported age of onset of myopia, which is subject to recall error. We also did not have data on time spent in near work, a known risk factor for myopia^43^, and therefore could not adjust for it in multivariable modes. We did, however, adjust for participant and parent education, which are likely to act as partial proxy measures of near work. Third, factor scores are indeterminate; there is no correct solution for any factor score calculated from a CFA model^44^. Thus, factor scores are imperfect estimates and this tends to result in a loss of statistical power^45^. Fourth, participants of the KYAMS were aware of the aims of the study; hence this could have introduced recall bias when participants were self-reporting their time spent outdoors in the sun. However, we validated these self-reported measures against objective measures. Fifth, at least half of the KYAMS participants were missing one or more measure of outdoor exposure. To mitigate any effect of missing data, we used full information maximum likelihood estimation, which produces estimates that are not biased in any particular direction in the presence of data that is missing at random. We additionally conducted a sensitivity analysis, which showed similar results. Last, due to the relatively small size of our study sample, we may have lacked power to detect some associations or may have overfit the data by adjusting for multiple covariates, although evidence show that as little as 2 and 10 subjects-per-variable are required for the unbiased estimation of linear^46^ and logistic^47^ regression coefficients, respectively. Strengths of our study include the use of multiple measures of past time spent outdoors in the sun to create sun indices, the long follow-up period, the assessment of time outdoors over a long period of time, and the measurement of refraction at an age when it has largely stabilised.

In summary, we did not find an effect of the Kidskin Study sun-exposure intervention on risk of myopia. Spending more time outdoors during childhood was associated with lower risk of myopia in young adulthood and spending more time outdoors during late adolescence and young adulthood was associated with a lower risk of myopia onset during this time. Spending more time outdoors in childhood, adolescence and early adulthood are likely to have long-term, potentially life-long, benefits in preventing myopia.

## Supplementary Information

Supplementary Information.

## Data Availability

The datasets generated during and/or analysed during the current study are available from the corresponding author on reasonable request.
