# Supplementary material for: Time spent outdoors in childhood is associated with reduced risk of myopia as an adult
Source: Sci Rep. 2021 Mar 18;11:6337. doi: 10.1038/s41598-021-85825-y (PMC7973740; doi:10.1038/s41598-021-85825-y)
Supplement: Supplementary file 1 — Supplementary Information. [file 41598_2021_85825_MOESM1_ESM.docx]

**SUPPLEMENTARY MATERIAL**

Time spent outdoors in childhood is associated with reduced risk of myopia as an adult

**Authors:** Gareth Lingham^1^, Seyhan Yazar^1^, Robyn M Lucas^1,2^, Elizabeth Milne^3^, Alex W Hewitt^1,4,5^, Christopher J Hammond^6^, Stuart MacGregor^7^, Kathryn A Rose^8^, Fred K Chen^1^, Mingguang He^4,9^, Jeremy A Guggenheim^10^, Michael W Clarke^11^, Seang-Mei Saw^12^, Cathy Williams^13^, Minas T Coroneo^14^, Leon Straker^15^, David A Mackey^1^*

^1^Centre for Ophthalmology and Visual Science (incorporating Lions Eye Institute), The University of Western Australia, Perth, Australia

^2^National Centre for Epidemiology and Population Health, Research School of Population Health, Australian National University, Canberra, Australia

^3^Telethon Kids Institute, The University of Western Australia, Perth, Australia

^4^Centre for Eye Research Australia, Department of Ophthalmology and Surgery, University of Melbourne, Royal Victorian Eye and Ear Hospital, Melbourne, Australia

^5^Department of Ophthalmology, Menzies Institute of Medical Research, University of Tasmania, Hobart, Australia

^6^Section of Academic Ophthalmology, School of Life Course Sciences, King’s College London, London, UK

^7^Statistical Genetics Lab, QIMR Berghofer Medical Research Institute, Brisbane, Queensland, Australia

^8^Discipline of Orthoptics, Graduate School of Health, University of Technology Sydney, Sydney, Australia

^9^State Key Laboratory of Ophthalmology, Zhongshan Ophthalmic Center, Sun Yat-sen University, Guangzhou, China

^10^School of Optometry & Vision Sciences, Cardiff University, Cardiff, UK

^11^Metabolomics Australia, Centre for Microscopy, Characterisation and Analysis, The University of Western Australia, Perth, Australia

^12^Department of Epidemiology and Public Health, Yong Loo Lin School of Medicine, National University of Singapore, Singapore, Singapore

^13^Department of Population Health Sciences, Bristol Medical School, University of Bristol, Bristol, UK

^14^Department of Ophthalmology, University of New South Wales, Sydney, Australia

^15^School of Physiotherapy and Exercise Science, Curtin University, Perth, Australia

**Supplementary Material**

*De-seasonalizing 25-hydroxyvitamin D concentration*

Serum 25(OH)D concentration varies with season, we therefore used a cosine model to adjust 25(OH)D concentration for month of collection (deseasonalised).^1^ Vitamin D supplementation was simultaneously included as a covariate to adjust for its effect on 25(OH)D concentration, which is unrelated to outdoor exposure.

*Specification of Confirmatory Factor Analysis Model*

All indicator variables, except skin damage score, were square root-transformed prior to CFA analysis to obtain a more normal distribution. A quantile-quantile plot confirmed that the multivariate distribution of indicator variables was approximately normal.

To outline the initial structure of the CFA model, an exploratory factor analysis in the R package “psych” was conducted with maximum likelihood estimation, Promax transformation, and a “mixed” correlation to account for continuous and ordinal variables. Exploratory factor analysis indicated that the measures of time spent outdoors were best explained by two factors. Factor 1 was most strongly correlated with measures of childhood time spent outdoors and factor 2 was most strongly correlated with measures of more recent time spent outdoors (≥15 years of age; Supplementary Figure 1).

We then specified a CFA model with full information maximum likelihood estimation using the R package “lavaan”. Covariance terms were included between self-reported measures of time spent outdoors and between parent-reported measures as these variables are likely related through pathways other than outdoor exposure (e.g. measurement method). Indicator variables associated with factor/s at p<0.10 were retained in the model. Factor scores were generated from the final CFA models using the regression method, which produces normally distributed factor scores that range between -1 and 1 and have a mean of 0.^2,3^

**REFERENCES**

1. van der Mei IAF, Blizzard L, Ponsonby A-L, Dwyer T. Validity and Reliability of Adult Recall of Past Sun Exposure in a Case-Control Study of Multiple Sclerosis. *Cancer Epidemiol Biomarkers Prev.* 2006;15(8):1538-1544.

2. Estabrook R, Neale M. A Comparison of Factor Score Estimation Methods in the Presence of Missing Data: Reliability and an Application to Nicotine Dependence. *Multivariate Behav Res.* 2013;48(1):1-27.

3. DiStefano C, Zhu M, Mîndrilă D. Understanding and using factor scores: Considerations for the applied researcher. *Prac Assess Res Eval.* 2009;14(20):1-11.

**Supplementary Table 1** Descriptive statistics of measures of time spent outdoors

|  | Median | IQR | n (%) |
| --- | --- | --- | --- |
| Time spent outdoors (hrs/day) |  |  |  |
| Parent-report (8 years) | 1.9 | 1.1 – 3 | 270 (89.1%) |
| Parent-report (10 years) | 1.9 | 1.1 – 3.1 | 270 (89.1%) |
| Parent-report (12 years) | 1.7 | 1.0 – 2.8 | 257 (84.8%) |
| Self-reported (5-9 years) | 2.0 | 1.4 – 2.5 | 245 (80.9%) |
| Self-reported (10-14 years) | 2.0 | 1.5 – 2.9 | 245 (80.9%) |
| Self-reported (15-19 years) | 1.9 | 1.3 – 2.6 | 245 (80.9%) |
| Self-reported (20-26 years) | 1.7 | 1.1 – 2.6 | 246 (81.2%) |
| Self-reported current time spent outdoors | 1.6 | 0.9 – 2.4 | 261 (86.1%) |
| Deseasonalised 25(OH)D concentration (nmol/L) | 68.9 | 52.0 – 81.8 | 259 (85.5%) |
| Total CUVAF area (mm²) | 32.5 | 13.7 –51.6 | 294 (97.0%) |
| Naevus count – right arm | 20.0 | 8.0 – 33.0 | 303 (100%) |
| Yearly change in number of back naevi 6-12 years | 0.83 | 0.5 – 1.67 | 264 (87.1%) |
| Skin score | n | % | 295 (97.4%) |
| 1 | 48 | 16.27% |  |
| 2 | 107 | 36.27% |  |
| 3 | 94 | 31.86% |  |
| 4 | 46 | 15.59% |  |

**Supplementary Table 2** Univariable associations between objective measures of time in sun (column headings) and subjective or other objective measures of time in sun (row headings)

|  | 25(OH)D conc (nmol/L)^a,d^ | | Total CUVAF area (mm²)^a^ | | Skin damage score^c^ | | Right arm naevus count^b^ | | Annual change back nevi  6-12 y^b^ | | |
| --- | --- | --- | --- | --- | --- | --- | --- | --- | --- | --- | --- |
|  | Beta (95% CI) | p | Beta (95% CI) | p | Odds ratio  (95% CI) | p | Relative increase (95% CI) | p | Relative increase (95% CI) | | p |
| Time spent in the sun (hrs/day) |  |  |  |  |  |  |  |  |  |  | |
| Parent-report (8 years) | 2.40 (0.41, 4.40) | **0.02** | 3.03 (1.09, 4.97) | **0.002** | 1.16 (1.00, 1.34) | 0.05 | 1.02 (0.94, 1.11) | 0.59 | 1.09 (1.01, 1.18) | **0.02** | |
| Parent-report (10 years) | 2.40 (0.06, 4.74) | **0.046** | 2.85 (0.73, 4.97) | **0.009** | 1.19 (1.01, 1.41) | **0.04** | 1.08 (0.99, 1.18) | 0.07 | 1.08 (0.98, 1.18) | 0.09 | |
| Parent-report (12 years) | 2.43 (0.50, 4.35) | **0.01** | 1.62 (-0.20, 3.43) | 0.08 | 1.09 (0.96, 1.25) | 0.18 | 0.97 (0.91, 1.04) | 0.47 | 0.97 (0.89, 1.04) | 0.39 | |
| Parent-reported  (mean 8-12 years) | 4.33 (1.72, 6.94) | **0.001** | 4.25 (1.81, 6.69) | **0.001** | 1.30 (1.08, 1.57) | **0.005** | 1.02 (0.92, 1.13) | 0.68 | 1.08 (0.97, 1.19) | 0.16 | |
| Self-reported (5-9 years) | 1.41 (-1.72, 4.54) | 0.38 | 0.89 (-1.89, 3.66) | 0.53 | 1.10 (0.89, 1.36) | 0.37 | 0.97 (0.88, 1.08) | 0.58 | 1.01 (0.89, 1.13) | 0.92 | |
| Self-reported (10-14 years) | 2.70 (-0.57, 5.97) | 0.11 | 2.06 (-0.80, 4.92) | 0.16 | 1.09 (0.88, 1.36) | 0.44 | 1.01 (0.90, 1.12) | 0.92 | 1.01 (0.89, 1.14) | 0.89 | |
| Self-reported (15-19 years) | 5.01 (1.76, 8.26) | **0.003** | 4.18 (1.33, 7.02) | **0.004** | 1.09 (0.87, 1.35) | 0.48 | 1.02 (0.92, 1.14) | 0.69 | 0.98 (0.86, 1.11) | 0.75 | |
| Self-reported (20-26 years) | 4.36 (1.13, 7.58) | **0.001** | 4.56 (1.76, 7.35) | **0.002** | 1.17 (0.94, 1.45) | 0.16 | 1.04 (0.93, 1.16) | 0.53 | 0.99 (0.87, 1.12) | 0.90 | |
| Self-reported current time outdoors in sun | 2.27 (0.12, 4.42) | **0.04** | 2.73 (0.82, 4.64) | **0.006** | 1.15 (1.01, 1.32) | **0.04** | 0.99 (0.92, 1.07) | 0.83 | 1.04 (0.96, 1.12) | 0.37 | |
| Deseasonalised 25(OH)D  per 10nmol/L increase | NA | NA | 0.86 (-0.33, 2.05) | 0.16 | 1.03 (0.95, 1.13) | 0.46 | 1.03 (0.99, 1.07) | 0.27 | 1.03 (0.97, 1.08) | 0.32 | |
| Total CUVAF area (mm²) per 10mm² increase | 0.94 (-0.36, 2.23) | 0.16 | NA | NA | 1.06 (0.98, 1.16) | 0.17 | 1.03 (0.98, 1.07) | 0.24 | 0.97 (0.93, 1.02) | 0.30 | |
| Skin damage score  per 1 category increase | 1.41 (-1.92, 4.74) | 0.41 | 2.37 (-0.64, 5.39) | 0.12 | NA | NA | 0.99 (0.88, 1.11) | 0.84 | 1.19 (1.04, 1.35) | **0.009** | |
| Arm Naevus count per 10 naevus increase | 1.15 (-0.31 2.60) | 0.12 | 0.80 (-0.53, 2.14) | 0.24 | 0.99 (0.90, 1.10) | 0.88 | NA | NA | 1.14 (1.08, 1.19) | **<0.001** | |
| Annual change back nevi 6-12y | 1.29 (-1.36, 3.93) | 0.34 | -1.17 (-3.60, 1.21) | 0.34 | 1.19 (1.09, 1.32) | **0.004** | 1.20 (1.09, 1.31) | **<0.001** | NA | NA | |

CI: Confidence interval; 25(OH)D: 25-hydroxyvitamin D; CUVAF: Conjunctival ultraviolet autofluorescence; conc.: concentration

Column headings are the outcomes in the regression models, row headings are the explanatory variables

Skin damage score is an ordinal variable with higher score indicating worse skin damage

^a^Linear regression; ^b^Negative binomial regression – relative increase in number of naevi per unit increase, an increase >1 indicates more naevi

^c^Ordinal logistic regression – an odds ratio above 1 indicates higher odds of having worse actinic skin damage

^d^25(OH)D concentration deseasonalised according to month of collection and concurrently adjusted for vitamin D supplement use

**Supplementary Table 3** Standardized factor loadings and r-square statistics of the three specified confirmatory factor analysis (CFA) models

|  | Total sun index model | | Childhood sun index model | | Recent sun index model | |
| --- | --- | --- | --- | --- | --- | --- |
| Indicators | Standardized factor loading | R-squared | Standardized factor loading | R-squared | Standardized factor loading | R-squared |
| Parent 1997 | 0.45 | 0.20 | 0.47 | 0.22 | NA | NA |
| Parent 1999 | 0.44 | 0.19 | 0.49 | 0.24 | NA | NA |
| Parent 2001 | 0.39 | 0.15 | 0.24 | 0.06 | NA | NA |
| Skin score | 0.24 | 0.06 | 0.29 | 0.08 | NA | NA |
| CUVAF area | 0.41 | 0.17 | 0.43 | 0.18 | 0.38 | 0.15 |
| Back nevi | 0.13 | 0.02 | NA | NA | NA | NA |
| 25-D levels | 0.41 | 0.17 | NA | NA | 0.36 | 0.13 |
| Self 15-26y | 0.49 | 0.24 | NA | NA | 0.66 | 0.44 |
| Self current | 0.41 | 0.17 | NA | NA | 0.49 | 0.24 |

Standardized factor scores are the correlation between the indicator variable and the latent variable and the r-square is the square of the standardized factor loading. Note factor loadings will differ to the correlation between the indicator variable and the factor scores generated from the model due to the indeterminacy of factor scores (i.e. factor scores are only an estimate of the true latent variable). Parent: Parent-reported time spent outdoors. Self: self-reported time spent outdoors. 25-D: 25-hydroxyvitamin D. CUVAF: conjunctival ultraviolet autofluorescence. Y: years. Back nevi: average yearly change in number of nevi on the backs between ages 6-12 years. Skin score: skin damage score.

**Supplementary Table 4** Univariable associations between myopia or spherical equivalent and individual measures of time spent outdoors

|  | | Myopia | | | Mean Spherical Equivalent^a^ | | |
| --- | --- | --- | --- | --- | --- | --- | --- |
| Odds ratio (95%CI) | | | p | n | Beta (95% CI) | p | n |
| Intervention Group |  | |  | 303 |  |  | 282 |
| Control | Reference | |  |  | Reference |  |  |
| Moderate | 0.93 (0.51, 1.67) | | 0.80 |  | -0.07 (-0.58, 0.44) | 0.79 |  |
| High | 0.93 (0.50, 1.73) | | 0.82 |  | 0.03 (-0.51, 0.57) | 0.92 |  |
| Time spent outdoors (hrs/day) |  | |  |  |  |  |  |
| Parent-reported (8 years) | 0.94 (0.79, 1.12) | | 0.52 | 270 | 0.11 (-0.05, 0.26) | 0.17 | 251 |
| Parent-reported (10 years) | 0.70 (0.56, 0.87) | | **0.002** | 270 | 0.27 (0.11, 0.43) | **<0.001** | 253 |
| Parent-reported (12 years) | 0.81 (0.67, 0.98) | | **0.03** | 257 | 0.19 (0.05, 0.33) | **0.008** | 241 |
| Parent-reported (mean of 8-12 years) | 0.71 (0.55, 0.90) | | **0.005** | 296 | 0.33 (0.14, 0.51) | **<0.001** | 276 |
| Self-reported KYAMS sun calendar (mean 15-26 years) | 0.83 (0.63, 1.10) | | 0.20 | 246 | 0.29 (0.05, 0.52) | **0.02** | 230 |
| Self-reported current time outdoors (KYAMS) | 0.81 (0.66, 0.99) | | **0.04** | 261 | 0.25 (0.11, 0.40) | **0.001** | 243 |
| Deseasonalised 25(OH)D  per 10nmol/L increase | 0.99 (0.89. 1.10) | | 0.84 | 259 | 0.04 (-0.05, 0.13) | 0.36 | 243 |
| Total CUVAF area (mm²) per 10mm² increase | 0.86 (0.77, 0.96) | | **0.008** | 292 | 0.16 (0.07, 0.25) | **<0.001** | 277 |
| Skin score per 1 category increase | 1.06 (0.81, 1.39) | | 0.65 | 293 | -0.0003 (-0.23, 0.23) | 0.99 | 277 |
| Average rate of change of back naevi 6-12 years |  | |  |  |  |  |  |

25(OH)D: 25-hydroxyvitamin D. CUVAF: conjunctival ultraviolet autofluorescence

^a^18 participants missing post-cycloplegic spherical equivalent data (n=11) or had prior refractive surgery (n=7) and 3 participants with moderate- to high-hyperopia excluded as outlier

**Supplementary Table 5** Sensitivity analysis using data with complete sun exposure measures only: n=126 for total, n=143 for recent and child

|  | Myopia^a^ | | | | |  | Spherical Equivalent^b^ | | | |
| --- | --- | --- | --- | --- | --- | --- | --- | --- | --- | --- |
|  | Univariate |  | | | Multivariate^c^ | | Univariate (n=126) |  | Multivariate^c^ | |
|  | OR (95% CI) | p | | | OR (95% CI) | p | Beta (95% CI) | p | Beta (95% CI) | p |
| **All participants (n=46 with myopia)** | |  | | |  |  |  |  |  |  |
| Total sun exposure | 0.85 (0.67, 1.08) | 0.18 | | | 0.87 (0.66, 1.14) | 0.31 | 0.17 (-0.05, 0.39) | 0.13 | 0.07 (-0.16, 0.31) | 0.55 |
| Child sun exposure | 0.79 (0.67, 0.93) | **0.005** | | | 0.80 (0.67, 0.97) | **0.03** | 0.20 (0.06, 0.35) | **0.008** | 0.06 (-0.09, 0.21) | 0.42 |
| Recent sun exposure | 0.89 (0.78, 1.02) | 0.10 | | | 0.93 (0.80, 1.07) | 0.31 | 0.19 (0.07, 0.31) | **0.003** | 0.12 (0.007, 0.24) | **0.04** |
| **Myopia onset <15 years (n=17) vs no myopia** | | | |  |  |  |  |  |  |  |
| Total sun exposure | 0.80 (0.56, 1.16) | 0.24 | | | 0.51 (0.47, 1.39) | 0.44 | 0.16 (-008, 0.40) | 0.20 | 0.04 (-0.21, 0.30) | 0.76 |
| Child sun exposure | 0.71 (0.56, 0.90) | **0.005** | | | 0.75 (0.55, 1.04) | 0.08 | 0.21 (0.04, 0.37) | **0.01** | 0.05 (-0.11, 0.21) | 0.53 |
| Recent sun exposure | 0.84 (0.66, 1.07) | 0.15 | | | 0.87 (0.64, 1.18) | 0.38 | 0.17 (0.03, 0.31) | **0.02** | 0.08 (-0.05, 0.21) | 0.25 |
| **Myopia onset ≥15 years (n=29) vs no myopia** | | |  | |  |  |  |  |  |  |
| Total sun exposure | 0.81 (0.67, 0.98) | **0.03** | | | 0.80 (0.65, 0.98) | **0.03** | 0.05 (-0.04, 0.14) | 0.24 | 0.03 (-0.07, 0.13) | 0.56 |
| Child sun exposure | 0.83 (0.67, 1.02) | 0.08 | | | 0.78 (0.61, 0.99) | **0.04** | -0.02 (-0.12, 0.08) | 0.72 | -0.03 (-0.14, 0.08) | 0.56 |
| Recent sun exposure | 0.88 (0.74, 1.04) | 0.12 | | | 0.86 (0.72, 1.04) | 0.11 | 0.09 (0.007, 0.17) | **0.03** | 0.07 (-0.01, 0.16) | 0.10 |

^a^Logistic regression; ^b^Linear regression

one person missing recent sun exposure; three people with moderate- to high-hypermetropia were excluded from analyses of spherical equivalent

^c^Adjusted for age, sex, university education, outdoor occupation, parental myopia, parental education, Caucasian/non-Caucasian race and Kidskin Study intervention group

All effect sizes per a 0.1 unit change in factor


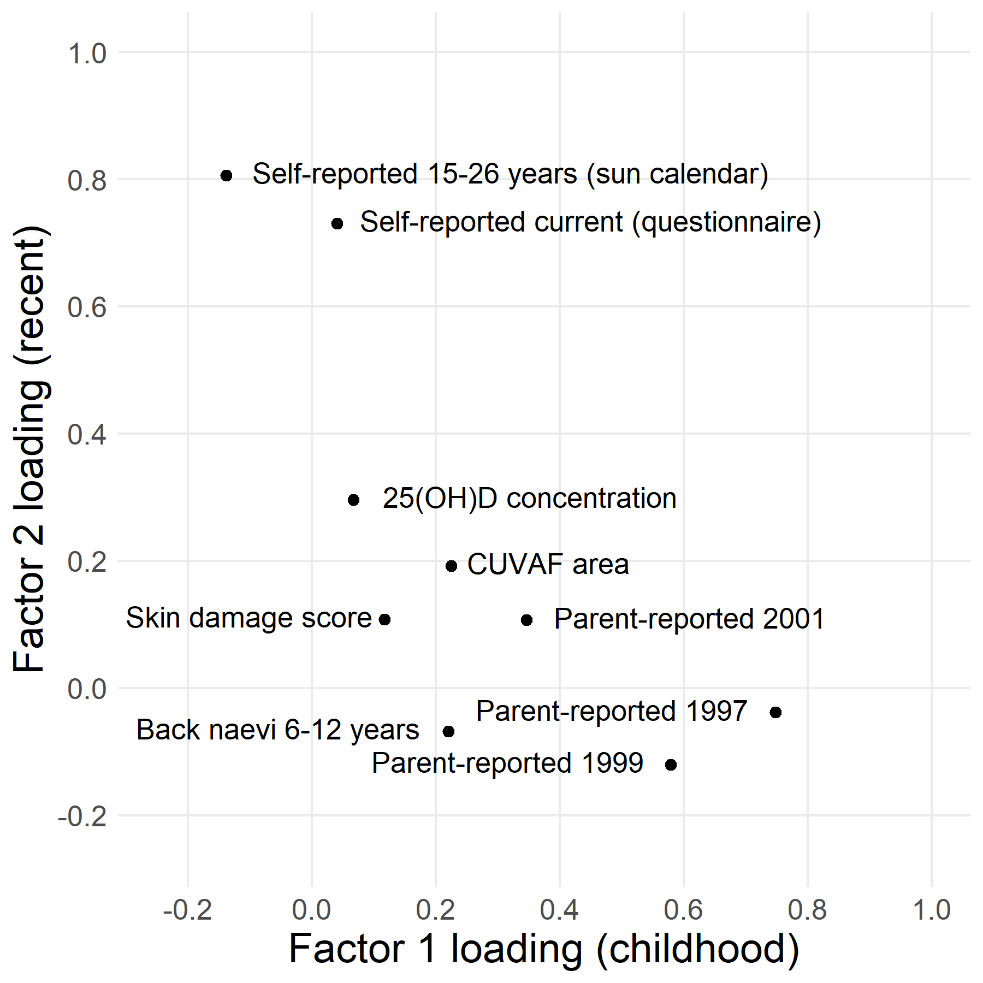


**Supplementary Figure 1** Results of exploratory factor analysis showing correlation between the indicator variables and each of factors 1 and 2
